# miR-17 and -20a Target the Neuron-Derived Orphan Receptor-1 (NOR-1) in Vascular Endothelial Cells

**DOI:** 10.1371/journal.pone.0141932

**Published:** 2015-11-23

**Authors:** Irene Sambri, Javier Crespo, Silvia Aguiló, Diego Ingrosso, Cristina Rodríguez, José Martínez González

**Affiliations:** 1 Centro de Investigación Cardiovascular (CSIC-ICCC), IIB-Sant Pau, Barcelona, Spain; 2 Department of Biochemistry, Biophysics & General Pathology, School of Medicine & Surgery, Second University of Naples, Naples, Italy; China Medical University, TAIWAN

## Abstract

Neuron-derived orphan receptor-1 (NOR-1) plays a major role in vascular biology by controlling fibroproliferative and inflammatory responses. Because microRNAs (miRNAs) have recently emerged as key players in the regulation of gene expression in the vasculature, here we have investigated the regulation of NOR-1 by miRNAs in endothelial cells. Computational algorithms suggest that NOR-1 could be targeted by members of the miR-17 family. Accordingly, ectopic over-expression of miR-17 or miR-20a in endothelial cells using synthetic premiRNAs attenuated the up-regulation of NOR-1 expression induced by VEGF (as evidenced by real time PCR, Western blot and immunocitochemistry). Conversely, the antagonism of these miRNAs by specific antagomirs prevented the down-regulation of NOR-1 promoted by miR-17 or miR-20a in VEGF-stimulated cells. Disruption of the miRNA-NOR-1 mRNA interaction using a custom designed target protector evidenced the selectivity of these responses. Further, luciferase reporter assays and seed-sequence mutagenesis confirmed that miR-17 and -20a bind to NOR-1 3’-UTR. Finally, miR-17 and -20a ameliorated the up-regulation of VCAM-1 mediated by NOR-1 in VEGF-stimulated cells. Therefore, miR-17 and -20a target NOR-1 thereby regulating NOR-1-dependent gene expression.

## Introduction

The vascular remodeling underlying coronary heart disease (CHD) and other vascular diseases involves complex regulatory networks whose activity should be tightly regulated. We and others have recently involved NR4A receptors in vascular remodeling [1–5]. The NR4A subfamily of nuclear receptors consists of three closely related members: Nur77, Nurr1 and NOR-1 (Neuron-derived Orphan Receptor-1; NR4A3) [6]. These nuclear receptors regulate diverse biological processes and have been implicated in a variety of high-incidence human pathologies including obesity, diabetes, cardiovascular disease and cancer [6–10]. NR4A receptors seem to be constitutively active, ligand-independent transcription factors [11], which act as early-response genes up-regulated by a variety of stimuli [6].

All 3 NR4A receptors are over-expressed in atherosclerotic plaques from human coronary arteries [1–3,12,13], and in arteries from animal models subjected to vascular injury [1,4,5]; however, only NOR-1 has positively been involved in vascular injury-induced neointimal hyperplasia [4,5]. In vascular cells, NOR-1 expression is induced by growth factors, cytokines and molecules with mitogen-like activity such as lipoproteins or thrombin [1,13–17]. NOR-1 regulates the spreading, migration and proliferation of vascular cells [1,13–18], participates in the survival response of endothelial cells to hypoxia [19], and modulates vascular inflammation [20,21]. This transcription factor controls gene expression acting through a response element (NBRE) present in the promoter of its target genes [6,22]. NOR-1 activity is mainly dependent on its expression levels, and substantial progress has been made in the knowledge of the extracellular cues that up-regulate NOR-1; however, its post-transcriptional regulation deserves further investigation. In this regard, recently, a DNA-dependent protein kinase (DNA-PK) posttranscriptional mechanism that phosphorylates and stabilizes NOR-1 has been reported [23].

MicroRNAs (miRNAs) are small (~22-nucleotide) non-coding RNAs that regulate gene expression at a post-transcriptional level by binding to the target mRNAs, leading either to degradation or to translational repression [24]. Due to their imperfect base-pairing with targets, miRNAs have the capacity to regulate many target mRNAs, therefore acting as global regulators of gene expression. In the last years a myriad of studies have evidenced the crucial role of miRNAs in the cardiovascular system and cardiovascular diseases [25–27]. Recently, NR4A receptors, particularly Nurr1, have been identified to be miRNA targets in different cells and tissues [28–30]. However, despite the growing relevance of NR4A receptors in vascular biology and cardiovascular diseases, to our knowledge, only a previous study has reported the regulation of one of these receptors by miRNAs in vascular smooth muscle cells [31]. Herein, we demonstrate that two members of the miR-17-92 cluster (miR-17 and miR-20a) bind to the human NOR-1 mRNA thereby modulating the endothelial expression of genes dependent on this transcription factor.

## Material and Methods

### Cell culture

Human umbilical vein endothelial cells (HUVEC; Lonza) were cultured in medium M199 (Gibco) supplemented with 20 mM HEPES pH 7.4 (Gibco), 30 μg/ml endothelial growth factor supplement (Sigma-Aldrich), 2 mM glutamine (Gibco), 1 mM pyruvate (Gibco), 100 μg/ml heparin (Sigma), 20% fetal calf serum (FCS, Biological Industries), and antibiotics (0.1 mg/ml streptomycin, 100 U/ml penicillin G; Gibco). The cells were used between passages 2 and 5. HUVEC were seeded in multiwell plates and were maintained under standard culture conditions (21% O_2_, 5% CO_2_, 95% humidity) until subconfluence. Then, cells were arrested overnight by incubation with medium containing 10% FCS. Finally, cells were stimulated with VEGF-A (100 ng/ml 2 h; R&D Systems). All the procedures were approved by the Reviewer Institutional Committee on Human Research of the Hospital of Santa Creu i Sant Pau (Comité Ético de Investigación Clínica del Hospital de la Santa Creu i Sant Pau) that conforms to the Declaration of Helsinki and written informed consent from donors was obtained.

### miRNA target Prediction

The bioinformatic analysis of miRNA predicted targets was determined by using three different algorithms [32]: TargetScan (http://www.targetscan.org/), that predicts biological targets of miRNA by searching for the presence of conserved 8mer and 7mer sites that match the seed region (nucleotides 2–7 at the 5’ end segment of the miRNA) of each miRNA; PicTar (http://pictar.mdc-berlin.de/) that predicts miRNA targets by searching for pair-wise alignments that are conserved across species, and miRanda (http://www.microrna.org/) that calculates free energy from miRNA-mRNA heteroduplex formation.

### Transient transfection of premirs, antagomirs and target protector

HUVEC were transfected with 100 nM precursor molecules mimicking miR-17 and miR-20a (pre-miR-17 and pre-miR-20a, Ambion by Life Technologies Corporation), 200 nM antagomirs (anti-miR-17 and anti-miR-20a, Applied Biosystems) and/or 500 nM miScript NOR-1 target protector (Qiagen) as indicated. A scramble sequence (Scr, Ambion by Life Technologies Corporation) was used as a control. Cells were transiently transfected using Lipofectamine RNAiMAX reagent (Life Technologies Corporation) according to the manufacturer’s protocol.

### Real time PCR

Total RNA was isolated using the RNAeasy kit (Qiagen) according to the manufacturer’s recommendations. RNA integrity was determined by electrophoresis in agarose gels and was quantified by a NanoDrop 1000 Spectrophotometer (Thermo Scientific). Total RNA (1 μg) was reverse-transcribed using the High Capacity cDNA Archive kit (Applied Biosystems, AB) and random hexamers. mRNA levels were assessed by real-time PCR on an ABI PRISM 7900 sequence detector (AB). TaqMan^™^ gene expression assays-on-demand (AB) were used for human NOR-1 (Hs00175077_m1) and vascular cell adhesion molecule-1 (VCAM-1) (Hs00365486_m1). The results were normalized by TATA binding protein (Hs99999910_m1). To analyze miR-17 and miR-20a total RNA was isolated with the mirVana^™^ miRNA kit (Life Technologies) and gene-specific TaqMan probes were employed for miR-17 (AB, 002309) and miR-20a (AB, 000580). The expression levels of miR-17 and miR-20a were calculated using the ΔΔCT method with RNU6B (AB, 001093) as the endogenous control.

### Western blot analysis

HUVEC were washed with PBS and lysed with a lysis buffer containing 0.5% SDS in 10 mM Tris–HCl (pH 7.4) and 1 mM ortovanadate. Protein concentration was measured by the BCA protein assay^™^ and proteins were resolved by SDS-PAGE and electrotransferred onto Immobilon polyvinylidene diflouride membranes (Millipore). Western blot analysis was performed using antibodies against NOR-1 (clone 1E11, Abnova) or VCAM-1 (sc-1504-R, Santa Cruz Biotechnology). Detection was performed using the appropriate horseradish peroxidase-conjugated antibody (Dako) and a chemiluminescent detection system (Supersignal West Dura^™^, Pierce). The size of detected proteins was estimated using protein molecular-mass standards (Fermentas). β-actin (ab8226, Abcam) was used as a loading control.

### Confocal analysis

HUVEC, cultured in glass-bottom dishes (Willco Wells B.V.), were transfected with pre-miR-17 or pre-miR-20a in the presence or in the absence of their respective inhibitors. Then cells were arrested overnight and stimulated with VEGF as described above. Cell monolayers were fixed with a 4% paraformaldehyde solution and were processed for immunocytochemistry. After permeabilization and blocking, cells were incubated with the primary antibody for 1 hour at room temperature. After washing, Alexa Fluor 663 conjugated immunoglobulin (Molecular Probes) was used as secondary antibody. Controls incubated with non-immune γ-globulin and without the primary antibody were included in all procedures. Finally, cells were mounted with ProLong^™^ mounting medium (Molecular Probes) and analyzed by confocal microscopy (Leica TCS SP2-AOBS).

### Reporter vector design

For luciferase reporter experiments a 508 bp fragment containing part of NOR-1 3‘-UTR was amplified by PCR from human cDNA using primers: forward 5’-CTAGTCTAGATGGGCCTCCAGCGCATCTTC-3’ (*Xba*I restriction site is underlined) and reverse 5’-ACGCGTCGACCCTGGCGAACAACCCTTGGC-3’ (*Sal*I restriction site is underlined). This fragment was inserted into the pGL3 control vector with a TK promoter (kindly provided by Dr. Giulio Piluso, Dept. of General Pathology and Oncology, S.U.N.) immediately downstream from the luciferase stop codon by *Xba*I/*Sal*I digestion (Fermentas) generating the pGL3/NOR1-3’UTR reporter plasmid. The NOR-1 3’-UTR seed sequence was mutated using the QuickChange II Site-directed mutagenesis kit (Stratagene) and the pGL3/NOR1-3’UTR vector as a template according to the manufacturer's instructions. Mutations were introduced with the following pairs of primers: 5'-CTGCTGGGATAGCATTGTCCAAAA***AC***GCTTTGTTAGCAATTTCTTAGAAA-3' and 5'-TTTCTAAGAAATTGCTAACAAAGC***GT***TTTTGGACAATGCTATCCCAGCAG-3' (changes introduced are indicated in italic and bold). Changes introduced by mutagenesis were confirmed by DNA sequencing.

### Dual luciferase reporter assay

HUVEC were transfected with the pGL3/NOR1-3’UTR reporter plasmid or the empty construct, used as a control, using Lipofectamine LTX reagent (Invitrogen) according to the manufacturer’s protocol. Briefly, transient transfections were performed in subconfluent cells seeded in six-well plates using 1 μg/well of the luciferase reporter plasmid, 0.03 μg/well of pRL-CMV (Promega) as an internal control, and 3 μl of Lipofectamine LTX. The activities of firefly and Renilla luciferases were determined in cell lysates using the Dual-Luciferase^®^ Reporter Assay System (Promega) and a luminometer (Orion I, Berthold Detection Systems) according to the manufacturer. Results were expressed as the ratio of firefly to renilla activity. The experiments were performed in triplicate.

### Statistical analysis

Data are expressed as mean ± SD (unless otherwise stated). Significant differences were established by Student's t-test or one-way ANOVA, according to the number of groups compared, using the GraphPad Instat program (GraphPad Software V2.03) (GraphPad Software Inc.). In the latter case, when significant variations were found, the Tukey–Kramer multiple comparison test was applied. Differences were considered significant at *p* < 0.05.

## Results

### Bioinformatic analysis of miRNA binding sites in the 3’-UTR of NOR-1 mRNA

**Fig 1:**
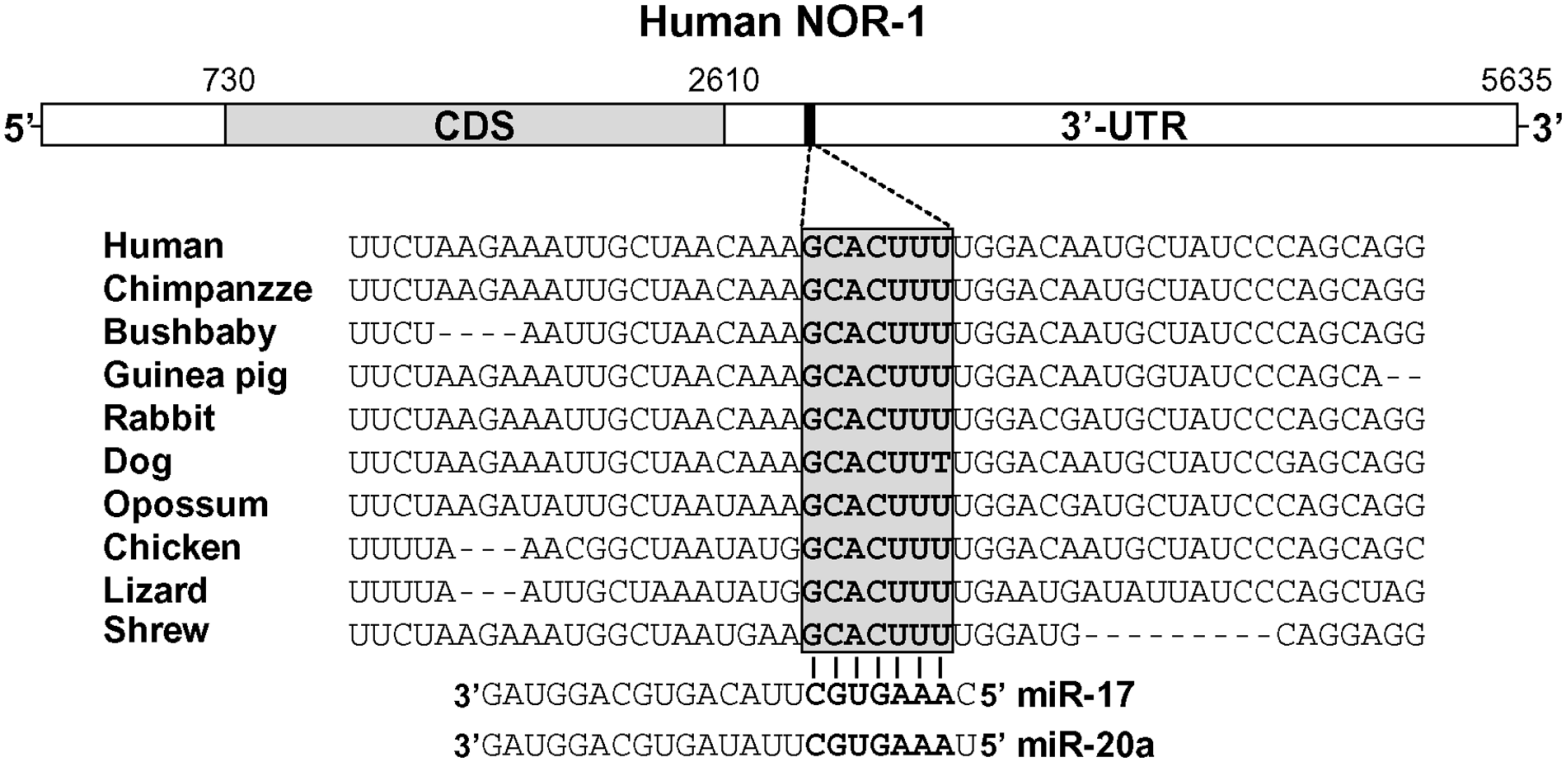
NOR-1 transcript contains a miR-17/-20 putative binding site in the 3’-UTR. Schematic representation of the human NOR-1 3’-UTR transcript (NM_006981) showing the highly conserved miR-17/-20 binding site among species (positions 2894–2900 in NM_006981 sequence). The seed sequence of miR-17/-20 (nt 2–10) is indicated in bold.

To investigate the potential regulatory properties of miRNAs on NOR-1 expression, miRNA binding sites in the human NOR-1 3’-UTR were examined by an *in silico* analysis using three of the most widely utilized algorithms (TargetScan, PicTar, and miRanda), which are based on different prediction criteria. Only miRNA binding sites independently predicted by the three applied algorithms would be considered. With this premise, we identified one binding site with a perfect complementarity to the seed region of the miR-17 and -20a (Fig 1). The miR-17/20 target site is a 7mer which is conserved within the NOR-1 3’-UTR across species (Fig 1).

### miR-17 and -20 regulate NOR-1 expression

**Fig 2:**
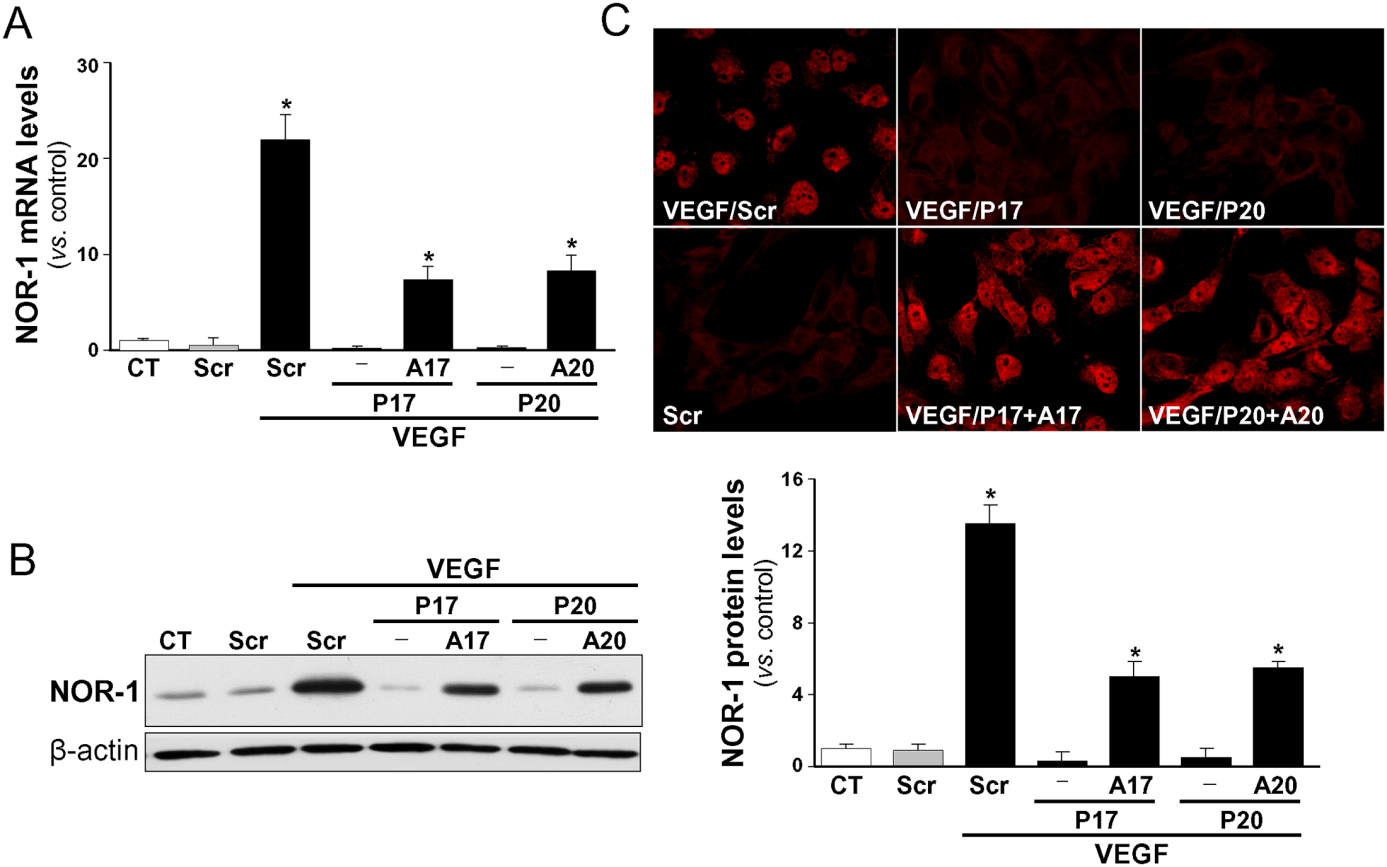
miR-17 and -20a regulate the expression of NOR-1. HUVEC were transfected with precursors of either miR-17 or miR-20a (P17 or P20) or a scramble sequence (Scr) in the presence or in the absence of their corresponding specific antagomirs (A17 or A20). Cells were stimulated with VEGF (100 ng/ml, 2 h). NOR-1 expression was assessed by real-time PCR **(A)**, Western-blot **(B)** and immunocytochemistry **(C)**. Results are expressed as mean ± SD from at least n = 4. (*p*<0.05: *, *vs*. untransfected cells [Control, CT] or cells transfected with Scr).

To assess whether NOR-1 could be a genuine miRNA-17/20 target, miR-17 and -20a were individually over-expressed in endothelial cells. Because the low expression of NOR-1 in resting vascular cells could hamper further analysis, we performed our studies in VEGF-stimulated cells in which NOR-1 expression is strongly induced [16,33]. HUVEC were transfected with precursors of either miR-17 or miR-20a followed by VEGF stimulation (100 ng/ml, 2 h). As shown in Fig 2A, both pre-miR-17 and -20a drastically inhibited the increase on NOR-1 mRNA levels triggered by VEGF with a similar efficiency. Likewise, western-blot analysis evidenced that the over-expression of these miRNAs blocked the VEGF-mediated up-regulation of NOR-1 protein levels (Fig 2B). Conversely, inhibition of endogenous miR-17 and -20a with specific antagomirs significantly counteracted the inhibitory effect of pre-miR-17 and -20a on the VEGF-induced NOR-1 up-regulation (both mRNA and protein levels) (Fig 2A and 2B). Similar results were obtained in confocal microscopy analysis (Fig 2C). As expected, VEGF stimulation of HUVEC results in an increased NOR-1 nuclear staining that was prevented by transfection of pre-miR-17 or pre-miR-20a, while cotransfection with their specific antagomis restore a similar phenotype to that observed in VEGF-treated cells. In agreement with previous results [34], VEGF was able to up-regulate the endogenous expression of miR-17 (Control cells *vs*. VEGF treated cells: 1.00 ± 0.23 *vs*. 2.27 ± 0.45) and pre-miR-20a (Control cells *vs*. VEGF treated cells: 1.00 ± 0.24 *vs*. 7.92 ± 0.34). Therefore, taken together these data suggest that the early-gene NOR-1 could be a bona fide target gene of the miR-17 family after stimuli that transiently increase its expression.

### Interference of the NOR-1 3’-UTR seed region blocks the miR-17/20-dependent regulation of NOR-1 expression

**Fig 3:**
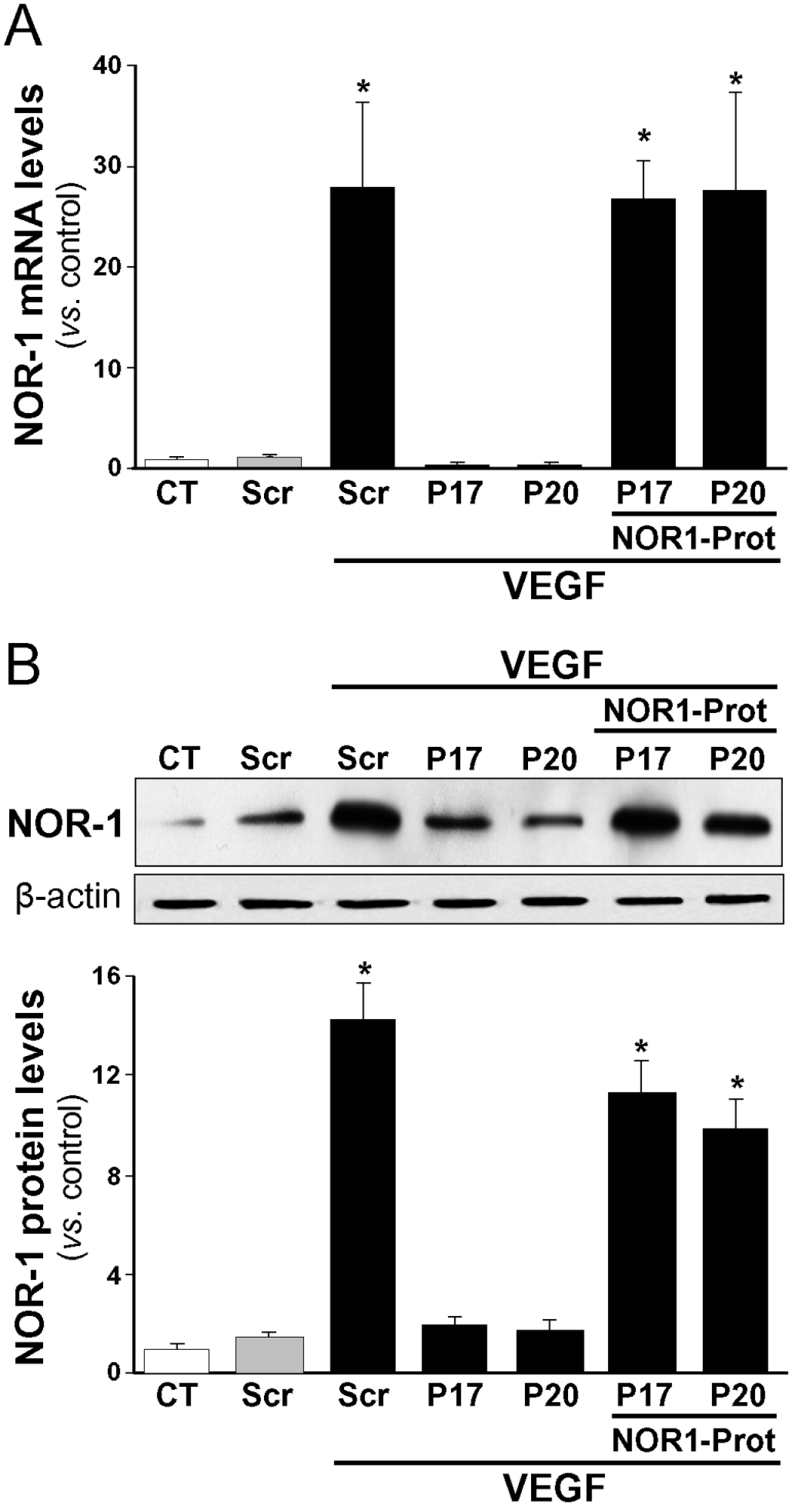
A protector sequence against the NOR-1 3’-UTR seed region prevents the regulation by miR-17 and -20. HUVEC were transfected with precursors of either miR-17 or miR-20a (P17 or P20) or a scramble sequence (Scr) in the presence or in the absence of a miScript target protector that blocks the NOR-1 3’-UTR seed sequence recognized by the miR-17 and -20 (NOR1-Prot). Then cells were stimulated with VEGF (100 ng/ml, 2 h). NOR-1 expression was assessed by real-time PCR **(A)** and Western-blot **(B)**. Results are expressed as mean ± SD from at least n = 4. (*p*<0.05: *, *vs*. untransfected cells [Control, CT] or cells transfected with Scr).

To assure that the observed responses are mediated by the selective binding of members of the miR-17 family to the NOR-1 3’-UTR region and not by indirect or unspecific mechanisms, a custom designed target protector was used. A target protector binds to the miRNA-binding site in a particular target gene blocking the access of a specific miRNA to this site, while leaving the regulation of other targets of the same miRNA unaffected. As observed in Fig 3, pre-miR-17 and -20a blocked the VEGF-mediated induction of NOR-1 mRNA and protein levels and interestingly, this effect was prevented by a target protector of the NOR-1 3’-UTR site.

### miR-17 and -20 bind to the NOR-1 3’-UTR putative seed sequence

**Fig 4:**
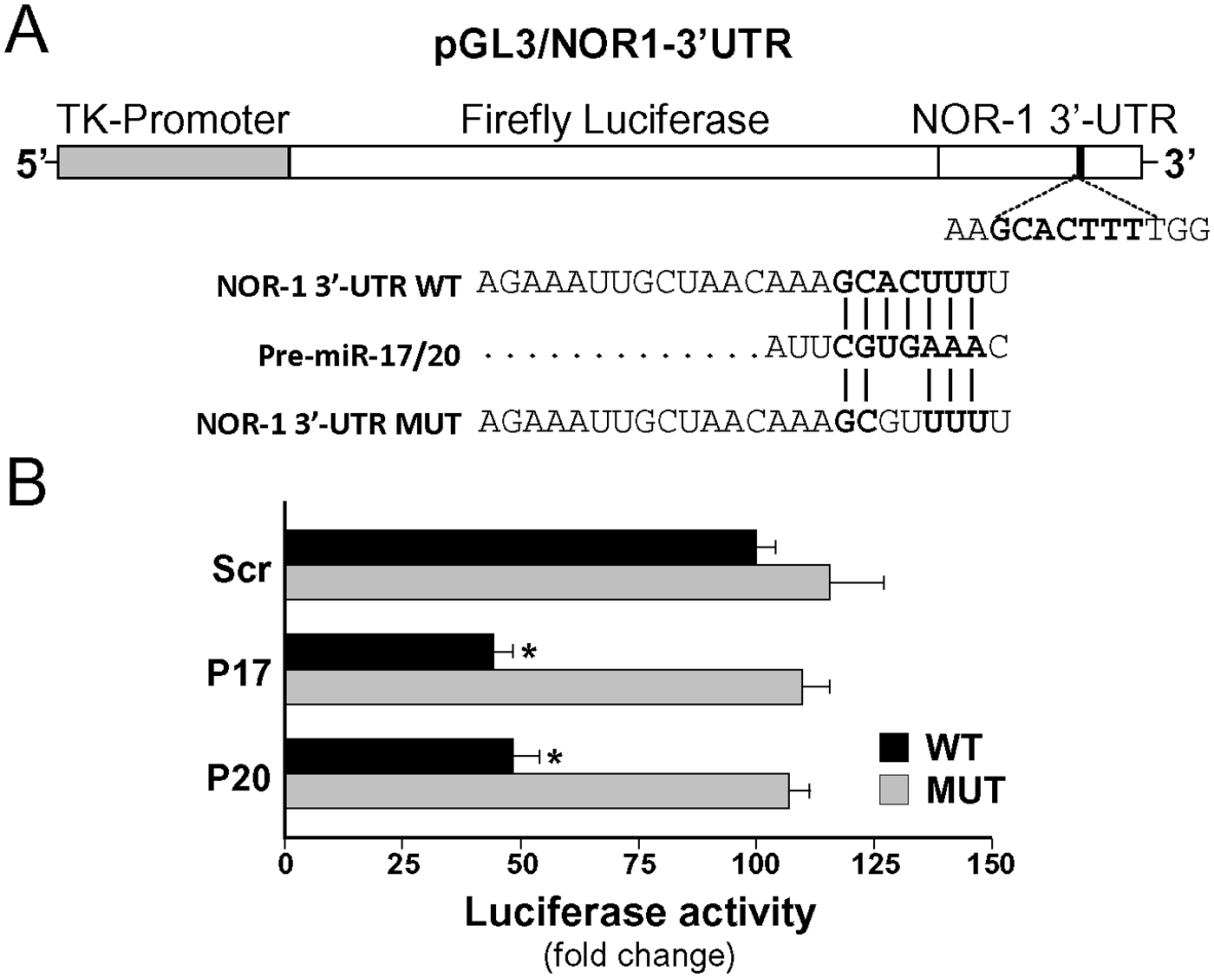
miRNA-17 and -20a bind to NOR-1 3’-UTR. **(A)** Schematic representation of the pGL3 luciferase reporter construct containing a 508 bp fragment of the NOR-1 3’-UTR region cloned downstream of the firefly luciferase gene (pGL3/NOR1-3’UTR). The sequences corresponding to the wild type miR-17/-20 binding site in this region (3’-UTR WT) and its mutated form (3’-UTR MUT) are shown below. **(B)** Luciferase activity in cell lysates from HUVEC transfected with the WT or the mutated luciferase reporter construct and cotransfected with precursors of either miR-17 or miR-20a (P17 or P20) or a scramble sequence (Scr). Firefly and Renilla luciferase activities were determined under each experimental condition. Results, normalized by Renilla activity. Results are expressed as mean ± SD from at least n = 4. (*p*<0.05: *, *vs*. cells transfected with Scr or with the MUT construct).

To further confirm the direct regulation of NOR-1 by the miR-17 and -20a we performed luciferase reporter assays in which the NOR-1 3’-UTR was cloned immediately downstream of the firefly luciferase open reading frame in the pGL3TK vector as previously reported [35]. A construct with an altered (mutated) seed sequence was also analyzed. Constructs containing the wild-type or the mutant NOR-1 3′-UTR (Fig 4A) were co-transfected with pre-miR-17 or pre-miR-20a into HUVEC, and luciferase activity was measured. Over-expression of both miR-17 and -20a reduced luciferase activity driven by the wild-type construct, while the mutant construct was unresponsive to these miRNAs (Fig 4B). These findings confirm that members of the miR-17 family bind the NOR-1 3’-UTR region.

### miR-17 and -20 target NOR-1 affecting NOR-1-dependent gene regulation

**Fig 5:**
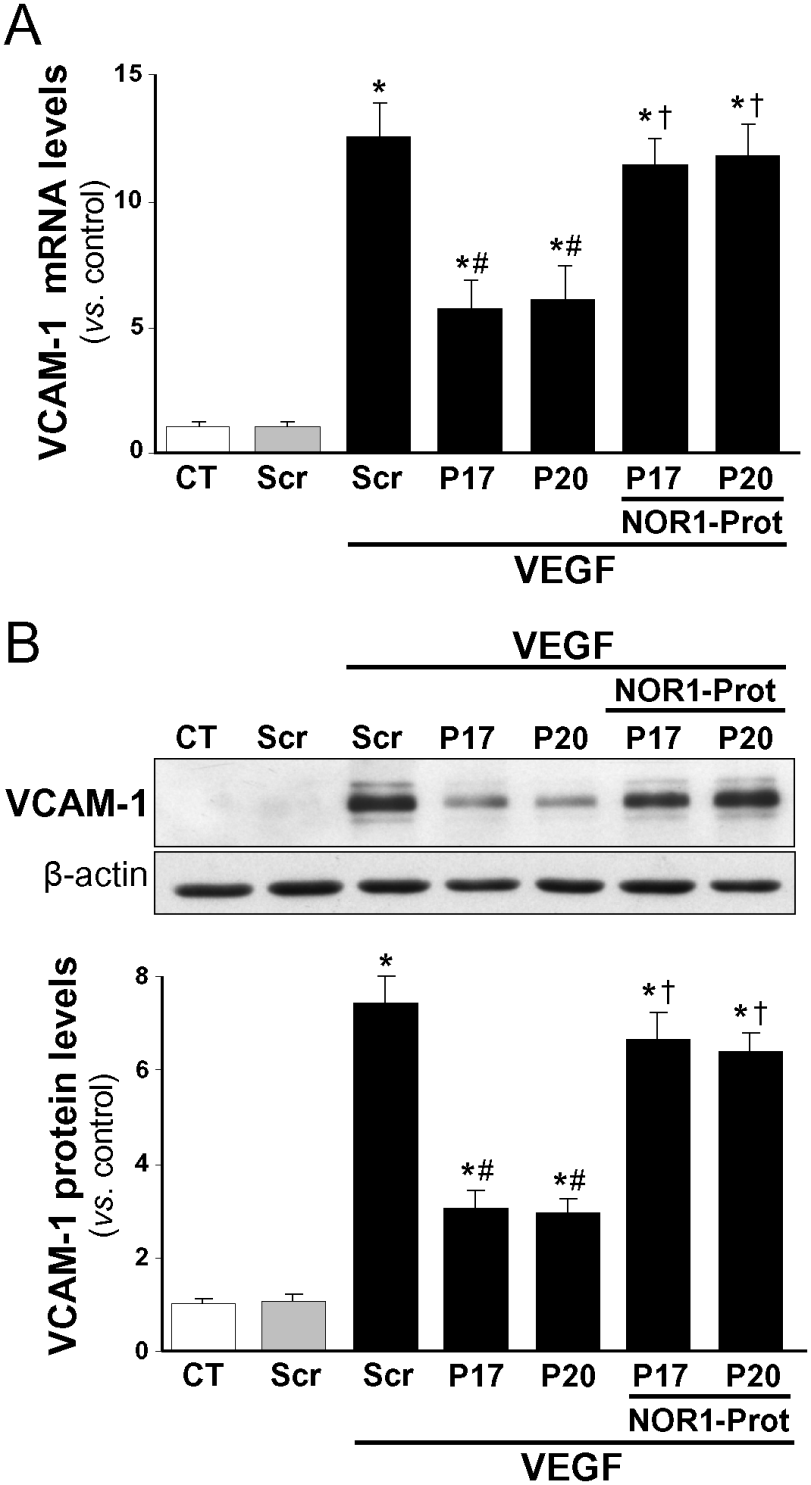
The NOR-1 protector prevents the down-regulation of VCAM-1 promoted by miR-17 and -20. HUVEC were transfected with precursors of either miR-17 or miR-20a (P17 or P20) or a scramble sequence (Scr) in the presence or in the absence of a miScript target protector that blocks the NOR-1 3’-UTR seed sequence recognized by the miR-17 and -20a (NOR1-Prot). Then cells were stimulated with VEGF (100 ng/ml, 2 h). VCAM-1 expression was assessed by real-time PCR **(A)** and Western-blot **(B)**. Results are expressed as mean ± SD from at least n = 4. (*p*<0.05: *, *vs*. untransfected cells [Control, CT] or cells transfected with Scr; #, *vs*. cells transfected with Scr and stimulated with VEGF; †, *vs*. cells exposed to the same condition but without NOR1-Prot).

Next we explored whether miR-17 and -20a could modulate the ability of NOR-1 to control gene expression. We analyzed the effect of miR-17 or miR-20a on the expression levels of VCAM-1, a NOR-1 target gene recently identified in human endothelial cells [20]. HUVEC were transfected with precursors of either miR-17 or miR-20a, as indicated above, and were stimulated with VEGF (100 ng/ml, 2 h). VEGF increased VCAM-1 expression (mRNA and protein levels), and both pre-miRs significantly reduced VCAM-1 expression, while the NOR-1 target protector prevented such down-regulation (Fig 5).

## Discussion

MicroRNAs are major regulators of global gene expression. Further, these small molecules are released by cells and can be detected in the blood. Most of the circulating microRNAs regulated in CHD patients are expressed by endothelial cells [36]. Indeed, endothelial microRNAs are both critical gene-regulatory factors controlling vascular function and inflammation and emerging biomarkers and therapeutic tools for cardiovascular diseases [37]. Moreover, the nuclear receptor NOR-1 has been recognized as a key transcription factor regulating the migration, proliferation and survival of endothelial cells [16,17,19], as well as vascular inflammation [20,21]. While multiple studies have described the up-regulation of NOR-1 expression by extracellular stimuli in vascular cells, mechanisms involved in the post-transcriptional regulation of this early-response gene are poorly understood. Here, we show that miR-17 and miR-20a target this transcription factor thereby modulating NOR-1-dependent up-regulation of VCAM-1.

The combined analysis with three different miRNA target site prediction databases consistently identified a miR-17/20 target site in the NOR-1 3’-UTR conserved among several species. Cross-species conservation of a potential binding site often underscores a genuine target gene, thus, *in silico* analysis strongly suggest NOR-1 as a novel target for miR-17 family. Accordingly, this bioinformatic prediction was experimentally tested. We have demonstrated that the over-expression of individual members of the miR-17 family (miR-17 and miR-20a) in endothelial cells significantly down-regulates NOR-1 mRNA and protein levels in VEGF-stimulated cells, while specific antagomirs significantly prevented this effect, suggesting that miR-17 and -20a destabilize NOR-1 transcripts and prevent its translation. Furthermore, our data from 3’-UTR luciferase reporter assays and those from experiments using a target protector to interfere the NOR-1 3’-UTR site indicate that this miRNA family regulates NOR-1 specifically, via the predicted seed sequence of NOR-1 3’-UTR region, and independently of the regulation that miR-17/20 could exert on other target genes. Finally, we also identified one binding site for miR-17 and -20a in the 3’UTR of other member of the NR4A family (Nurr1, NR4A2), and preliminary experimental data suggest the potential regulation of Nurr1 by these miRNAs in VEGF-induced HUVEC (data not shown). Therefore, our results demonstrate that NOR-1 is targeted by miR-17/20 and suggest that further studies could increase the repertoire of immediate early genes targeted by the miR17-92 cluster in vascular cells.

The miR-17-92 cluster is a polycistronic miRNA that yields six individual mature miRNAs, including miR-17, 18a, 19a, 20a, 19b, and 92a. Based on the sequence homology and seed conservation, the mir-17-92 cluster is subdivided into four miRNA families, one of them is the miR-17 family composed by miR-17 and miR-20a [38]. These miRNAs are well conserved in vertebrates [39], and play a role in regulating the physiological functions of many cell types [38]. Although miR-17-92 is considered an “oncomir” cluster, the biological effects of miR-17-92 individual components seem to be highly dependent on the cell type. miR-17/20 are deregulated in cardiovascular diseases. In fact, reduced plasma levels of miR17 have been described in patients with CHD and therefore this miRNA has been proposed as a potential biomarker for this pathology [36]. Interestingly, since this family is expressed in both endothelial cells and VSMC [40] it could play a relevant role regulating target genes in the vasculature. Several studies support an inhibitory effect of miR-17 on cell migration, but also inhibiting cell proliferation and the secretion of a subset of proinflammatory cytokines, at least in part, through the repression of the AIB1 gene [41–43]. Concerning endothelial cells, miR-17/20 have shown to inhibit cell sprouting, cell migration and exhibit a cell-intrinsic antiangiogenic activity [44–46]. Interestingly, this contrast with the well-documented role of NOR-1 as a transcription factor positively involved in cell sprouting, cell migration and cell proliferation [1,13–17]. Thus, the regulation of NOR-1 by the miR-17 family is consistent with the functions described for this transcription factor and this miRNA family in endothelial cells. In endothelial cells NOR-1 is induced by VEGF [16,33], a cytokine that up-regulates the expression of the adhesion molecule VCAM-1 [47] a target gene of NOR-1 [20]. miR-17 and -20a ameliorated the up-regulation of VCAM-1 in VEGF-stimulated cells. The additional involvement of other transcription factors in the induction of VCAM-1 promoted by VEGF (such as NFκB) [47], would explain the partial effect exerted by the premiRNAs. NOR-1 target protector prevented the effect of the premiRNAs indicating the involvement of NOR-1 in the regulation of VCAM-1. Interestingly, although VEGF primarily up-regulates NOR-1 through a transcriptional mechanism [16,33] it is also able to induce the expression of miR-17 and -20a [34] which ultimately negatively modulate NOR-1 mRNA stability/translation. In this regard, increasing results indicate that operating in concert, transcriptional and post-transcriptional mechanisms mediated by miRNAs contribute to the quick and transient up-regulation of immediate early genes [48].

In summary, the tight control of inflammatory and vascular remodeling processes would require the coordinated modulation of multiple structural genes through regulatory mechanisms affecting both gene expression and translation. Increasing evidence suggest that individual members of the miR-17-92 cluster modulate biological processes in a cell-specific manner [44]. We show that two miRNAs of this cluster target NOR-1 thereby down-regulating downstream endothelial gene expression dependent on this transcription factor. Therefore, the activity of NOR-1 as a transcription factor would be positively regulated primarily at transcriptional level (as an early-response gene quickly and strongly induced by different extracellular stimuli), but also by post-transcriptional mechanisms involving specific cellular miRNAs that negatively affect NOR-1 mRNA stability and translation. This could be regarded as an example of how cells integrate different regulatory mechanisms on key master genes/proteins to coordinate gene expression in complex biological processes. Such integration of different although potentially synergic mechanisms, involving the miR 17–92 cluster, further underscore the central role of NOR-1 in vascular remodeling.
